# Editorial: Advances and trends in nutraceutical and functional plant-based food

**DOI:** 10.3389/fnut.2023.1168826

**Published:** 2023-03-13

**Authors:** Gengjun Chen, Marina Carcea

**Affiliations:** ^1^Department of Grain Science & Industry, Kansas State University, Manhattan, KS, United States; ^2^Council for Agricultural Research and Economics, Rome, Italy

**Keywords:** plant-based food, bioactive nutrients, delivery system, functional properties, bioavailability, novel food processing, nutraceuticals

The production of large amounts of animal-based foods (such as meat, fish, eggs, and milk) has been suggested as one of the negative impacts on global environmental sustainability. Traditional livestock production for food typically causes more pollution and water use, which cause higher greenhouse gas emissions and lead to greater losses in biodiversity. As a result of environmental, ethical, and health concerns, the market for plant-based food is expanding rapidly, and the food industry is creating a new generation of plant-based products to meet this demand. Plant-based foods are commonly classified into vegetables, cereals, legumes, fruits, and nuts, and their derived processed counterparts such as baked goods, pasta, breakfast cereals, vegetable preserves, fermented fruits and vegetables, meat analogs, plant-based emulsions, gels, oils, and other delivery systems. Algae and mushrooms are also being explored as sources of novel foods.

Compared to animal-based food, there has been increasing scientific research on plant-based foods because of their potential sustainability and health-related benefits as foods rich in starch, proteins, lipids, dietary fiber, and phytonutrients. From microalgae to legumes, the potential health benefits of these vegetable ingredients are being explored and tested (1, 2). Cutting-edge techniques such as nanotechnology and encapsulation are being used to enhance the efficacy and bioavailability of components, making them even more attractive to consumers (3). The main challenge is to achieve nutrition and functionality in these plant-based foods using healthy and sustainable plant-derived ingredients with desirable appearance, flavor, sensory, and other physicochemical attributes (2, 3). It is also argued that large quantities of nutraceuticals and functional foods have to be consumed to achieve the alleged health benefits, despite the fact that the safety and health implications of plant bioactive compounds are not fully clear.

Eleven quality papers, eight research articles, and three review articles are published on this Research Topic. The following topics were investigated: “*Citrus peel flavonoid extracts—potential beneficial bioactivities and regulation of intestinal microecology in vitro*” (Li et al.); the role of macelignan in inhibiting Tau phosphorylation and Aβ aggregation in the cell model (Gu et al.); the potential benefits of using soybean press cake as an effective substrate for the commercial production of conjugated linoleic acid and eicosapentaenoic acid by *Bifidobacterium lactis* (Azari et al.); chrysanthemum flavonoids' main components, luteolin and luteoloside, and their potential hypolipidemic mechanism (Sun et al.); the potential antidepressant effects of chlorella and lion's mane mushroom complex as a dietary supplement (Chou et al.); the improvement of maize grain filling through novel high planting density and varying nitrogen application rate (Ren et al.); the assessment of golden-flowered tea (*Camellia nitidissima* Chi) fractions and their anti-non-small cell lung cancer (NSCLC) effects *in vitro* and *in vivo* (Wang et al.); “*The nutritional value of the extrusion-processed, micronutrient-fortified corn snacks enriched with protein and dietary fiber*” (Shah et al.). The reviews include a summary of the phytochemistry, pharmacology, and promising traditional uses of *Lagenaria siceraria* Molina (Standl.) fruit (Saeed et al.); ethnobotanical and pharmacological aspects of *Allium sativum* L. (garlic), with notes on its phytochemistry, ethnopharmacology, toxicological aspects, and clinical studies (Tudu et al.); and the advances in the study of *Syzygium aromaticum* (L) Merr. Perry, an aromatic plant in tropical regions worldwide, including information on its composition, phytochemistry, bioactive substances, and potential applications (Xue et al.).

In conclusion, the present Research Topic provides several examples of nutraceutical and functional plant-based foods and their applications. We hope it can provide new insights into innovative scientific understanding in this area and promote interest in further work and studies required for the development of nutraceuticals and functional plant-based foods.

## Author contributions

GC drafted the manuscript. GC and MC provided critical review and insight and revised the final version of the editorial. All authors contributed to the article and approved the submitted version.
